# A bibliometric analysis of non-coding RNA studies in acute pancreatitis

**DOI:** 10.1097/MD.0000000000037486

**Published:** 2024-03-22

**Authors:** Xiaodong Zhu, Kunrong Liu, Xiping Tang, Guozhong Chen

**Affiliations:** a Guangxi University of Chinese Medicine, Nanning, China; b The First Affiliated Hospital of Guangxi University of Chinese Medicine, Nanning, China; c Guangxi Medical University Affiliated Cancer Hospital, Nanning, China.

**Keywords:** acute pancreatitis, bibliometrics, hot spots, ncRNA, research trends

## Abstract

**Background:**

Non-coding RNA (ncRNA) is a type of RNA that does not code for proteins and plays a crucial role in the onset, progression, diagnosis, and therapy of acute pancreatitis. However, bibliometric, and visual analyses of studies on acute pancreatitis and ncRNA are lacking. This study seeks to provide a bibliometric overview of the knowledge structure and research hotspots of ncRNA in the field of acute pancreatitis research.

**Materials and methods:**

Literature search and collection of information in the field of ncRNA-related research in acute pancreatitis from 2000-2023 through the Web of Science Core Collection. Use CiteSpace and VOSviewer to visually analyze countries, institutions, authors, and keywords.

**Results:**

A total of 563 articles have been published in the field of ncRNA-related research in acute pancreatitis, and the number of publications in this field is gradually increasing. The largest number of publications was from China. Four clusters were produced by the co-occurrence cluster analysis of the top 89 keywords: studies of ncRNA in inflammation, autophagy, and apoptosis in acute pancreatitis; studies related to microRNA expression in pancreatic cancer among ncRNA; studies related to microRNAs as diagnostic and therapeutic markers in acute pancreatitis; and studies related to ncRNA in acute pancreatitis; The key words “injury,” “pathway” and “extracellular vesicles” are the key words of emerging research hotspots.

**Conclusion:**

In conclusion, ncRNA research in acute pancreatitis is an established discipline. Researchers can use the research hotspots and frontiers in this field as a guide for choosing their research direction.

## 1. Introduction

Acute pancreatitis (AP) is an inflammatory disease of the exocrine pancreas caused by biliary tract disease, alcohol, and hypertriglyceridemia, which can cause severe abdominal pain and multi-organ dysfunction and may result in pancreatic necrosis and persistent organ failure with a mortality rate of 1% to 5%.^[1,2]^ Pathological calcium signaling, mitochondrial dysfunction, premature activation of trypsinogen in islet cells and macrophages, endoplasmic reticulum stress, impaired unfolded protein response, and impaired autophagy are currently implicated in the pathogenesis of acute pancreatitis,^[3]^ however, the specific mechanisms of action and signaling pathways involved are not yet clear, and there is a lack of clear targets for diagnosis and treatment; therefore, exploring targeted therapies is a trend and hot spot in the field of acute pancreatitis research.

Non-coding RNA (ncRNA) is a type of RNA that does not code for proteins and plays an essential regulatory role in physiological processes, mediating signaling, gene expression and protein synthesis, on the other hand, abnormally expressed ncRNA is closely associated with the pathogenesis of many diseases.^[4]^ ncRNA includes micro RNA (miRNA), long non-coding RNA (lncRNA) and circular RNA (circRNA), which can control gene expression at the transcriptional or post-transcriptional level.^[5,6]^ Studies have demonstrated that ncRNAs participate in the pathological processes of AP, such as regulation of lipid metabolism,^[7]^ impact of the intestinal barrier^[8]^ and autophagy function,^[9]^ Intervention for pancreatic necrosis,^[10]^ etc. It can be seen that nc RNA plays an important role in the pathological process of acute pancreatitis, but there is a lack of systematic analysis of the mechanism of the role of ncRNA in acute pancreatitis disease, and the research progress and hotspots in this field.

Bibliometrics is a scientific quantitative research through mathematical and statistical methods, which can analyze the research trends in the field over a specific period of time and reveal the hot spots and frontiers of research by quantitative and qualitative analysis of the literature in the research field.^[11,12]^ In recent years, bibliometric studies have been used extensively to summarize the progress of clinical medicine and biomedical research.^[13–15]^ In addition, 2 data visualization tools, CiteSpace and VOSviewer, have been extensively utilized for bibliometric analysis.^[16]^ Therefore, bibliometrics is a good way to systematically analyze the relationship between ncRNAs and acute pancreatitis in order to dissect the current information on the number of publications, authors involved, research institutions, and keywords in this field of research.

It can be seen that with the investment and rapid development of a large number of scientific research, more and more ncRNAs have been discovered and studied, and the relationship between ncRNAs and acute pancreatitis has been revealed step by step, but currently there is a lack of bibliometric analysis and visualization of the literature related to ncRNAs and acute pancreatitis, which can systematically analyze the trends and hotspots in the field of research. Therefore, in this study, we searched the literature related to acute pancreatitis and ncRNA in Web of Science Core Collection (WoSCC) from 2000 to 2023 by bibliometric methods to analyze and summarize the current research status, development trend, and hotspots in this field, so as to provide new research ideas and directions for researchers and experts.

## 2. Materials and methods

### 2.1. Search strategy

**Figure 1.**
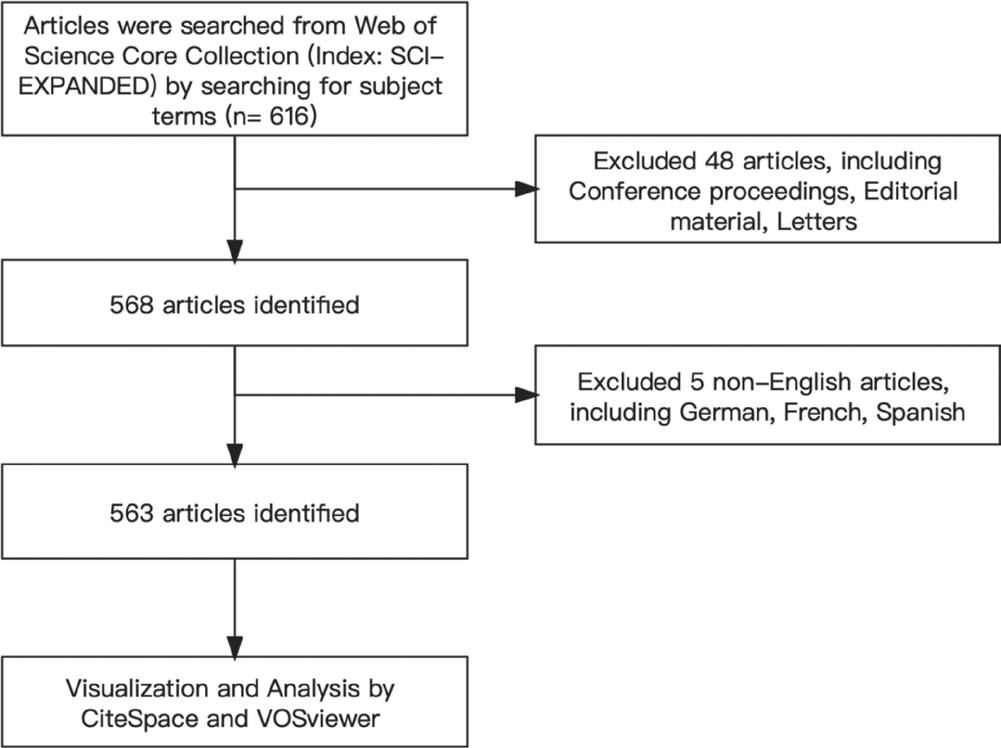
Flow chart of the literature study.

After we specified the search subject terms (Table 1), the search was performed at WoSCC with subject terms, version Science Citation Index Expanded (SCI-EXPANDED)-1999-present, time range: 2000-01-01-2023-05-10, article type set to thesis and review, language English, and the search results were exported in plain text for subsequent data and view network analysis. The particular procedure is depicted in Figure 1. (The data in this research comes from public databases, so it does not involve ethical approval.)

**Table 1:** Search terms.

Item	Search terms
#1	TS = (“Pancreatitis” OR “Pancreatitis, Acute Edematous” OR “Acute Edematous Pancreatitides” OR “Edematous Pancreatitides, Acute” OR “Edematous Pancreatitis, Acute” OR “Pancreatitides, Acute Edematous” OR “Acute Edematous Pancreatitis” OR “Pancreatic Parenchymal Edema” OR “Edema, Pancreatic Parenchymal” OR “Pancreatic Parenchymal Edemas” OR “Parenchymal Edema, Pancreatic” OR “Pancreatic Parenchyma with Edema” OR “Pancreatitis, Acute” OR “Acute Pancreatitis” OR “Acute Pancreatitides” OR “Pancreatitides, Acute” OR “Peripancreatic Fat Necrosis” OR “Fat Necrosis, Peripancreatic” OR “Necrosis, Peripancreatic Fat” OR “Peripancreatic Fat Necroses”)
#2	TS = (“Non-coding RNA” OR “microRNA” OR “microRNAs” OR “Circular RNA” OR “Circular RNAs” OR “long non-coding RNA” OR “long non-coding RNA” OR “lncRNA” OR “lncRNAs” OR “Small interfering RNA” OR “SiRNA” OR “Piwi-interacting RNA” OR “small nucleolar RNA” OR “ribosomal RNA” OR “transfer RNA” OR “small nuclear RNA” OR “tRNA-Derived Fragments” OR “tRNA halves” OR “miRNA” OR “circRNA” OR “tiRNA” OR “tRF” OR “miRNAs” OR “circRNAs” OR “miR*” OR “lnc*” OR “circ_*”)
#3	#1 AND #2

### 2.2. Data analysis

Use Excel to count relevant data, including annual publication volume, countries, institutions, authors, references, keywords, etc. Use VOSviewer (version 1.6.18) for visual web view production of country and institution collaboration, author and co-citation author collaboration, co-citation references, and keywords, In the view generated by VOSviewer, a node represents a project, and the node size and color indicate the number and classification of these projects, respectively. The width of the lines between nodes reflects the degree of collaboration or referencing of the projects.^[17,18]^ Use CiteSpace (version 6.2.2) for citation reference, keyword burst analysis, The basic parameter settings of this software refer to Zhang F’s article Settings.^[19]^

## 3. Results

### 3.1. General data

**Figure 2.**
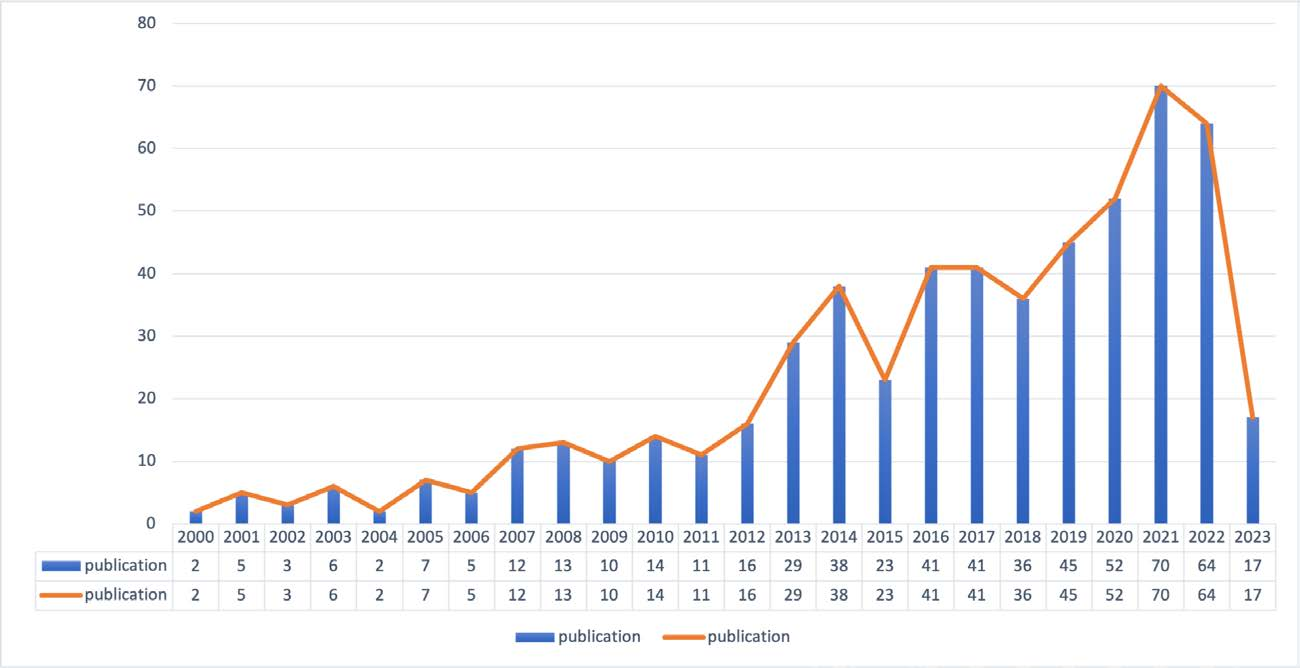
Annual posting volume view.

Based on our search strategy, a total of 563 articles were searched from 2000 to 2023, of which 510 (90.59%) were papers and 53 (9.41%) were reviews. The number of articles issued each year is shown in Figure 2. It can be seen that the number of publications increases year by year, indicating that a large amount of research is devoted to the field, which is getting more and more attention from researchers and experts.

### 3.2. Countries and institutions

**Figure 3.**
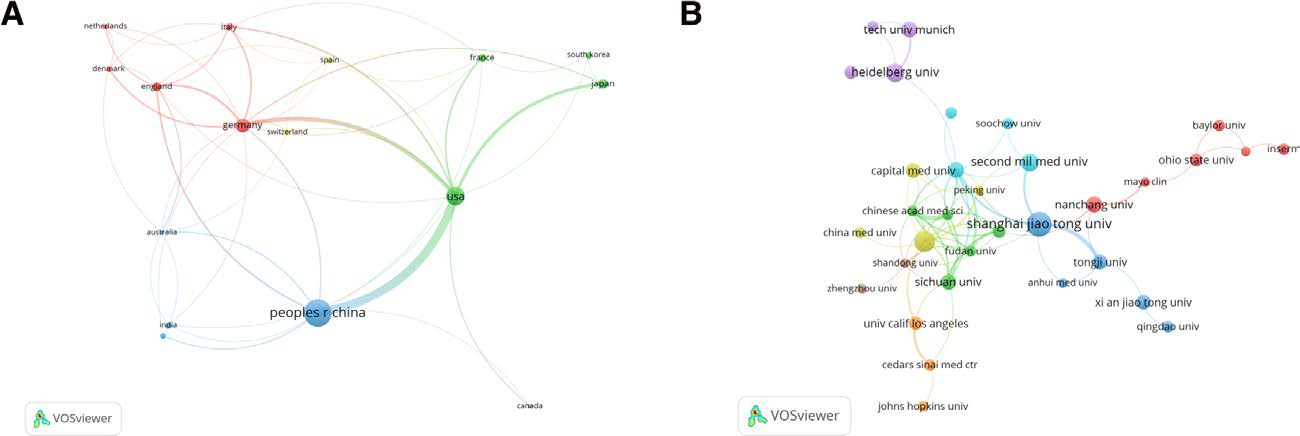
View of national (A) and institutional (B) collaborative networks in the field of non-coding RNA research in acute pancreatitis.

These 563 articles were published by 45 countries and 763 institutions. Table 2 displays the 10 nations with the highest number of published articles, and the most published country is China, followed by The United States and Germany. 17 countries with the number of articles published greater than or equal to 5 are analyzed in the network view using VOSviewer software, as shown in Figure 3A, which shows the cooperative relationship between countries, and it can be seen that China and the United States have a close cooperative relationship, in addition The United States and Germany and Japan also have a close cooperative relationship, but the cooperative communication between other countries is weak. Table 3 displays the 10 institutions with the greatest number of publications. 8 of these 10 institutions are from China and the other 2 are from Germany, and both countries are in the top 3 in terms of number of publications, indicating that China and Germany have more comprehensive and in-depth research in this field. As shown in Figure 3B, which shows the cooperative relationship between 39 institutions with the number of publications greater than or equal to 5, it can be seen that Shanghai Jiao Tong University, The Second Military Medical University, Tongji University and Nanjing Medical University, and Harbin Medical University and Peking University also have close collaborative relationships.

**Table 2:** Top 10 countries in terms of the quantity of published articles.

Rank	Country	Publications	Citations
1	China	294	6972
2	The United States	118	9015
3	Germany	61	2632
4	Japan	27	939
5	England	22	1054
6	France	16	746
7	Italy	12	1150
8	India	11	579
9	Spain	10	531
10	South Korea	10	160

**Table 3:** The top 10 institutions in terms of the quantity of published articles.

Rank	Institution	Publications	Citations
1	Shanghai Jiao Tong University	24	883
2	Harbin Medical University	19	281
3	Heidelberg University	15	802
4	The Second Military Medical University	14	747
5	Nanjing Medical University	12	676
6	Nanchang University	12	142
7	Technical University of Munich	12	370
8	Sichuan University	11	169
9	Tongji University	10	182
10	Capital Medical University	10	280

### 3.3. Authors and co-cited authors

**Figure 4.**
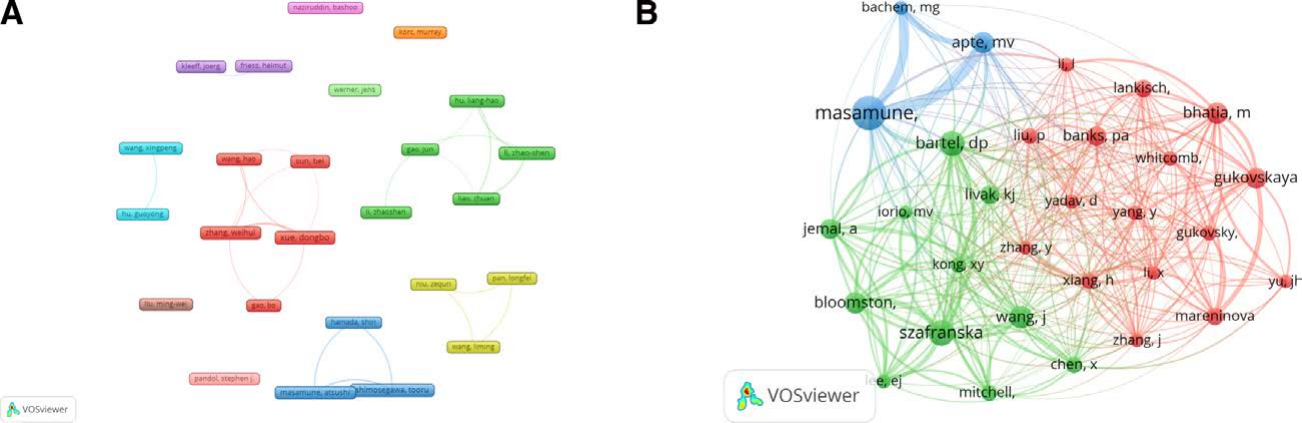
Network view of authors (A) and co-cited authors(B).

A total of 3528 authors were involved in these 563 publications. The top 10 authors are shown in Table 4, and the top 3 are Xue Dongbo, Zhang Weihui, and Masamune Atsushi. Although Xue Dongbo have the largest number of articles, the number of citations is not very high, and Gao Jun has the tenth largest number of articles, but the number of citations is the most in the top 10 authors, which indicates that they have a high reputation in the research field and are worth studying. Using VOSviewer software, we analyzed the collaboration view of the 25 authors with the number of articles greater than or equal to 5, as shown in Figure 4A, and we can see that the collaboration among the authors is not too close. A total of 15,478 authors were included among the co-cited authors in this research area, and 30 of them were cited more than 30 times in total, with Masamune Atsushi (98) having the most, followed by Anna E Szafranska (68), and David P Bartel (66). The view analysis of these 30 authors using VOSviewer software was divided into 3 clusters, as shown in Figure 4B, represented by 3 colors, Masamune, Atsushi and Minoti V Apte as the central blue cluster, Anna S Gukovskaya, Bhatia M and Banks PA as the central red cluster, Anna E Szafranska, Bartel DP, and Bloomston M were centered in the green cluster.

**Table 4:** The top 10 authors by quantity of published articles.

Rank	Authors	Publications	Citations
1	Xue Dongbo	10	94
2	Zhang Weihui	9	92
3	Masamune Atsushi	9	367
4	Shimosegawa Tooru	8	346
5	Li Zhaoshen	8	240
6	Sun Bei	8	168
7	Helmut Friess	7	237
8	Shin Hamada	6	221
9	Gao Jun	6	479
10	Bashoo Naziruddin	6	42

### 3.4. Co-citing references and citing outbreak references

**Figure 5.**
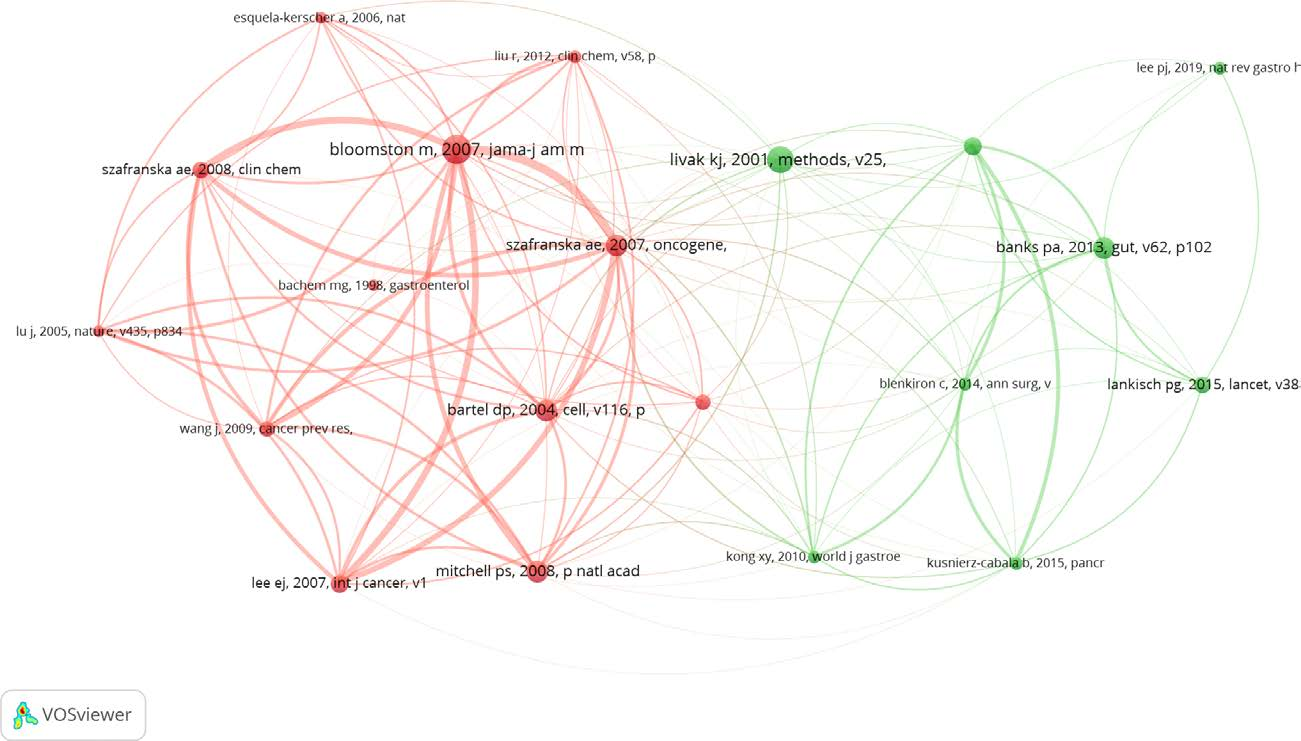
Network view of co-cited references.

Co-cited references are references that are cited together by the research field and can be considered as the research base of the field. Table 5 displays the 10 articles with the greatest frequency of citations. The most frequently co-cited article was “MicroRNA expression patterns to differentiate pancreatic adenocarcinoma from normal pancreas and chronic pancreatitis,” which examined the possibility that pancreatic cancer has distinct miRNA expression patterns that distinguish it from normal pancreas and chronic pancreatitis. We screened 20 articles with a co-citation frequency of more than 20 times using VOSviewer for view analysis, as shown in Figure 5.

**Table 5:** The 10 most co-cited references.

Rank	Co-cited reference	Citations	First author	Year	Magazine
1	MicroRNA expression patterns to differentiate pancreatic adenocarcinoma from normal pancreas and chronic pancreatitis	49	Bloomston M	2007	Jama-Journal of The American Medical Association
2	Analysis of relative gene expression data using real-time quantitative PCR and the 2(-Delta Delta C(T)) Method.	44	Livak KJ	2001	Methods
3	MicroRNA expression alterations are linked to tumorigenesis and non-neoplastic processes in pancreatic ductal adenocarcinoma	37	Szafranska AE	2007	Oncogene
4	MicroRNAs: Genomics, biogenesis, mechanism, and function	36	Bartel, DP	2004	Cell
5	Circulating microRNAs as stable blood-based markers for cancer detection	36	Mitchell, PS	2008	Proceedings of The National Academy of Sciences of The United States of America
6	Classification of acute pancreatitis-2012: revision of the Atlanta classification and definitions by international consensus	36	Banks, PA	2013	Gut
7	Expression profiling identifies microRNA signature in pancreatic cancer	31	Lee, EJ	2007	International Journal of Cancer
8	Identification of serum microRNAs as diagnostic and prognostic biomarkers for acute pancreatitis	30	Liu, P	2014	Pancreatology
9	Acute pancreatitis	29	Lankisch, PG	2015	Lancet
10	Analysis of microRNAs in pancreatic fine-needle aspirates can classify benign and malignant tissues	28	Szafranska, AE	2008	Clinical Chemistry

**Figure 6.**
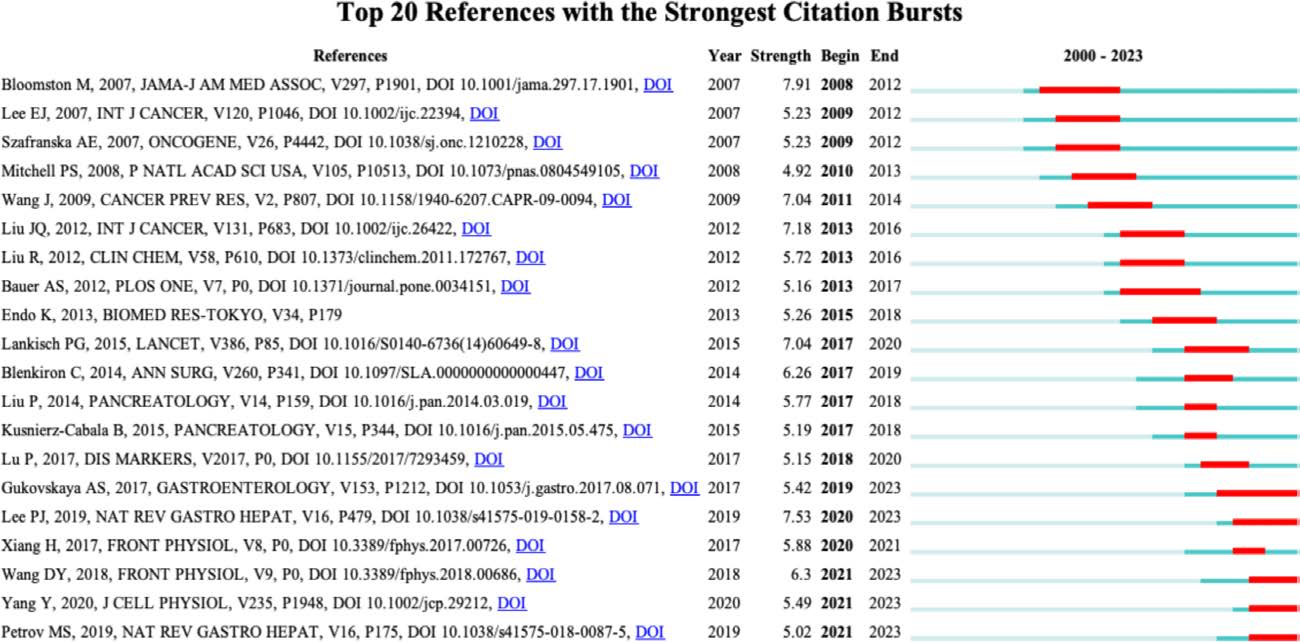
Outbreak of citation references.

We used CiteSpace to analyze 563 citation bursts and identified 20 articles with strong citation bursts, as shown in Figure 6. The top 3 articles with the greatest citation spikes were as follows: “MicroRNA expression patterns to differentiate pancreatic adenocarcinoma from normal pancreas and chronic pancreatitis” (intensity = 7.91), “New insights into acute pancreatitis” (intensity = 7.53), and “Combination of plasma microRNAs with serum CA19-9 for early detection of pancreatic cancer” (Intensity = 7.18).

### 3.5. Keyword analysis

**Figure 7.**
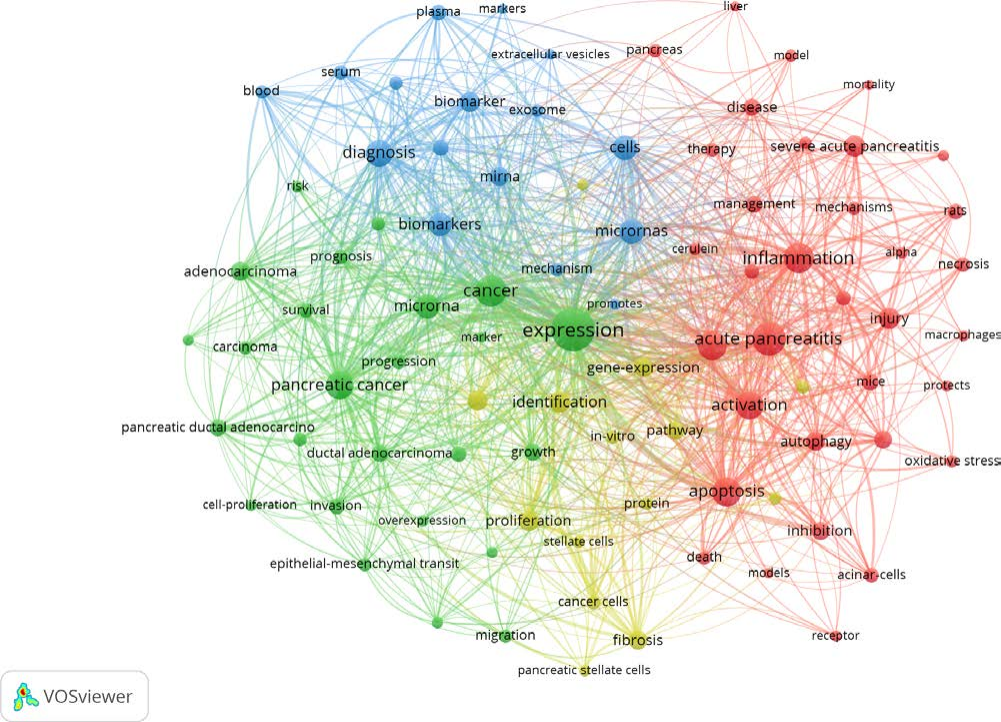
Keyword analysis view.

Keyword analysis was performed on 563 documents, and a total of 89 words appeared with a frequency greater than or equal to 10, and the top 20 keywords with the highest frequency are shown in Table 6. VOSviewer was used to construct a co-category view analysis of the screened 89 keywords, as shown in Figure 7, there are 4 clusters, with keywords depicted as node labels, the size of each node indicating its occurrence frequency, and the connection within 2 nodes indicating the co-occurrence relationship between 2 keywords. The red cluster mainly focuses on the study of non-coding RNA in the direction of inflammation, autophagy and apoptosis in acute pancreatitis; the green cluster mainly focuses on the study related to miRNA expression in pancreatic cancer among non-coding RNA; the blue cluster mainly focuses on the study related to miRNA as a diagnostic and therapeutic marker in acute pancreatitis; and the yellow cluster is related to the study of non-coding RNA expression in acute pancreatitis.

**Table 6:** The top 20 keywords with the greatest occurrence frequency.

Rank	Keyword	Counts	Cluster	Rank	Keyword	Counts	Cluster
1	expression	157	2	11	cells	53	3
2	acute pancreatitis	96	1	12	microrna	51	2
3	cancer	83	2	13	biomarkers	49	3
4	inflammation	80	1	14	identification	41	4
5	pancreatitis	74	1	15	severe acute pancreatitis	39	1
6	apoptosis	70	1	16	injury	38	1
7	pancreatic cancer	67	2	17	chronic pancreatitis	37	4
8	activation	67	1	18	proliferation	34	4
9	diagnosis	60	3	19	biomarker	34	3
10	micrornas	53	3	20	autophagy	31	1

**Figure 8.**
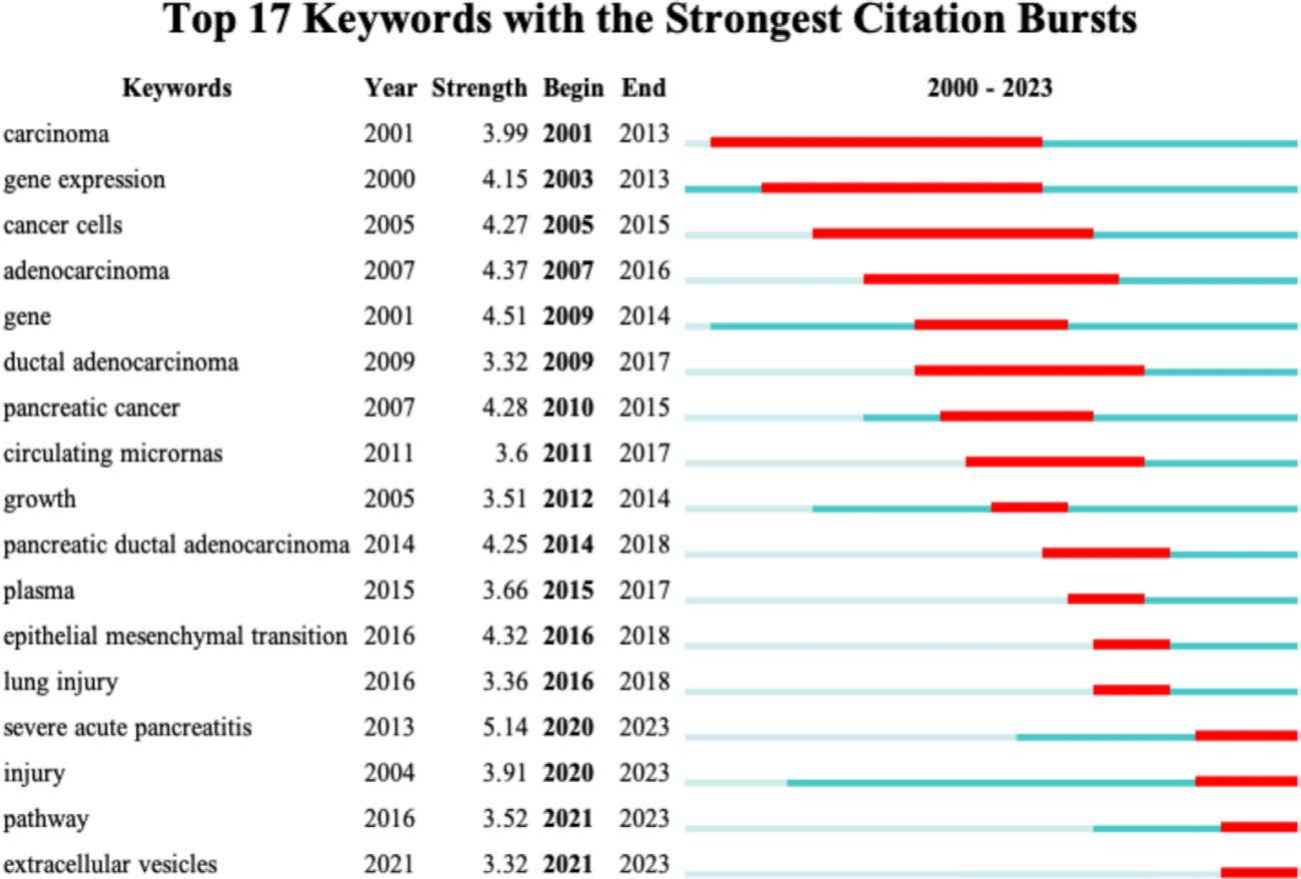
Keyword burst view.

Keyword outbursts respond to the research hotspots and trends in the research field, and the keyword outbursts in this study are shown in Figure 8. The 5 keywords with the strongest outbreak intensity were severe acute pancreatitis (SAP), gene, adenocarcinoma, cancer cells, and pancreatic cancer, which were obviously mainly focused on the direction of pancreatic-related cancer. The latest outbreak of keywords in the last 2 years are injury, pathway, and extracellular vesicles.

## 4. Discussion

### 4.1. Basic information

In this study, we used a bibliometric approach for the first time to analyze studies related to the field of non-coding RNA in acute pancreatitis. According to our search of 563 documents obtained from WoSCC, we discovered that the amount of publications in this field of study is increasing annually, which can be broken down into 3 stages: the initial period is from 2000 to 2006, with single-digit publications, the second stage is from 2007 to 2012, the number of publications exceeds 10 digits due to the accumulation of pertinent research base, and the third stage is from 2013 onwards, with the number of publications reaching 20 The third stage is after 2013, the number of articles reached 20, and increased rapidly year by year, indicating that more and more researchers have paid attention to the research field of non-coding RNAs in acute pancreatitis, and the most number of articles were published in 2021.

Based on the country perspective on the number of publications, China, the United States and France are the 3 countries with the highest number of publications, and China has the highest number of publications, far more than other countries, indicating that a large amount of funds and manpower are invested in the research of non-coding RNAs in acute pancreatitis. From the view of cooperation between countries, the cooperation between China and the United States, the United States, France, and Japan is relatively close, but the cooperation with other countries is not close enough, which is evidently detrimental to the exchange and cooperation in this study field and the growth of this research field, therefore, the exchange and cooperation between countries should be strengthened to promote the field forward. China is home to the majority of the top 10 institutions in terms of the quantity of publications, indicating that the research on the mechanism of non-coding RNA in acute pancreatitis is getting more and more attention from Chinese institutions. However, it is also evident from the institutional collaboration map that the collaboration among institutions still needs to be improved.

Xue, Dongbo, Zhang, Weihui and Masamune, Atsushi are the 3 authors with the highest number of publications in terms of author volume. Xue, Dongbo and Zhang, Weihui are jointly involved in screening and validating differentially expressed miRNAs in acute pancreatitis,^[20]^ and found that miRNAs play a role in the regulation of macrophage activation in pancreatitis,^[21]^ non-codified RNA was also found to have a role in apoptosis and autophagy in acute pancreatitis.^[22–24]^ Masamune, Atsushi investigated the alteration of miRNA expression profile during pancreatic stellate cell activation,^[25]^ and mainly engaged in pancreatic stellate cell related research.^[26–28]^ The above authors’ study has made a good accumulation for the study of non-coding RNA in acute pancreatitis. As for the co-cited authors of this study, Masamune, Atsushi, Anna E Szafranska and David P Bartel were the 3 most cited authors. Masamune, Atsushi focuses on the role of pancreatic stellate cells in pancreatic diseases,^[29]^ the expression of miRNAs in pancreatic stellate cells and autoimmune pancreatitis was also analyzed,^[25,30]^ it brings an important reference value for the subsequent study on the role of miRNA in acute pancreatitis. Anna E Szafranska assesses miRNA as a novel small RNA molecule with increasing therapeutic and diagnostic potential as an alternative analyte for gene expression studies in Formalin-Fixed Paraffin-Embedded Samples,^[31]^ moreover, the diagnostic potential of miRNAs in a clinical context was evaluated for the first time, providing a novel strategy for enhancing the diagnosis of pancreatic diseases.^[32]^ David P Bartel has made important contributions to the field of miRNA as a target recognition and regulatory function study.^[33–35]^ The above authors have a high academic influence and provide valuable citation references for subsequent studies.

In analyzing the co-cited references of this study, it was found that most of the 10 most frequently co-cited papers studied pancreatic cancer and miRNA correlation, which shows that this research direction is a trend and hot spot in this research field and is the basis of research on the correlation between acute pancreatitis and non-coding RNA. Bloomston M et al 2007 published the most frequently co-cited article,^[36]^ this article analyzes that pancreatic cancer may have a unique miRNA expression pattern that distinguishes it from normal pancreas and chronic pancreatitis, and that miRNA expression patterns can be used to assess the survival cycle of patients. Meanwhile, the reference with the highest citation burst intensity and the literature with the highest frequency of co-cited references were the same literature, which shows that the study of the correlation between pancreatic cancer and miRNA is currently the main topic in the field of non-coding RNA research in acute pancreatitis. In addition, references with the second highest citation burst intensity summarize the latest research advances in acute pancreatitis, with a focus on the disease’s pathophysiological mechanisms and clinical management strategies, providing researchers with new research ideas for studying.^[3]^ The reference with the third strongest citation burst is the analysis of miRNA that can effectively distinguish pancreatic cancer from non-pancreatic cancer and for the combination of miRNA and serum CA19-9 assay is more effective for early detection of pancreatic cancer.^[37]^ In summary, co-cited references and references with citation bursts are fundamental to this research area and provide evidence and guidance for future research.

### 4.2. Research hotspots and frontiers

Keyword analysis can classify and summarize the main knowledge structures and hotspots in the research field, and our keyword analysis facilitates us to quickly grasp the hotspots and trends in the field of non-coding RNA research on acute pancreatitis. In this study, we performed co-occurrence cluster analysis on 89 keywords and generated 4 main clusters, which are summarized below.

The first category is the study of non-coding RNA on inflammation, autophagy, and apoptosis in acute pancreatitis. Inflammatory response is an important factor in the occurrence, development, and evolution of acute pancreatitis, and identifying and blocking the inflammatory response is the key to treating pancreatitis. Non-coding RNAs serve a crucial role in the production of inflammatory factors and inflammatory cells in acute pancreatitis, for example, miRNA-93 can interfere with Toll-like receptor 4 (TLR4)/NF-κB signaling pathway to reduce inflammatory response by decreasing inflammatory factor production and improve apoptosis,^[38]^ miR-let-7a/let-7d/let-7f promotes inflammatory response, interleukin-20 production by M2-type macrophages,^[39,40]^ circRNAs utrophin reduce acute pancreatic inflammation and promote apoptosis via circ_UTRN/miR-320-3p/PTK2 axis,^[41]^ lncRNA NEAT1 inhibits apoptosis and inflammation in pancreatic follicular cells.^[42]^ Also, some non-coding RNAs have pro-inflammatory effects, while some non-coding RNAs exert anti-inflammatory effects; In the regulation of apoptosis, for example, microRNA-375 inhibits autophagy by targeting ATG7 and further promotes inflammation and apoptosis in glandular blast cells,^[43]^ upregulation of miR-374a-5p is beneficial for improving pancreatic follicular cell viability and inhibiting apoptosis and inflammation,^[44]^ miR-340-5p inhibits pancreatic follicular cell inflammation and apoptosis by targeting HMGB1;^[45]^ In autophagy-oriented studies, LncRNA-PVT1 exacerbates severe acute pancreatitis by promoting autophagy through the miR-30a-5p/Beclin-1 axis,^[46]^ miR-148a inhibits autophagy in acute pancreatitis through downregulation of IL-6/STAT3 signaling,^[47]^ in a mouse model of experimental pancreatitis, inhibition of the miR-155 pathway reduces impaired autophagy and improves prognosis.^[48]^ In addition, MiR-20b-5p regulates inflammation and apoptosis in severe acute pancreatitis by targeting AKT3 and interfering with autophagy.^[49]^ The above shows that non-coding RNA plays an important role in the pathological mechanism of acute pancreatitis, and there is a close undertaking relationship between autophagy, apoptosis, and inflammation, which deserves further in-depth study.

The second category is the research related to miRNAs in non-coding RNAs in the field of pancreatic cancer. miRNAs have been extensively studied in the direction of pancreatic cancer, such as by targeting particular signaling pathways, microRNA-195 inhibits the proliferation and invasion of pancreatic cancer cells,^[50]^ MicroRNA-374 regulates the proliferation and metastasis of human pancreatic cancer cells by targeting JAM-2,^[51]^ MicroRNA-34b inhibits pancreatic cancer metastasis by suppressing Smad3,^[52]^ MicroRNA-7 may serve as a potential biomarker for assessing the prognosis of pancreatic cancer.^[53]^ In addition, aberrant expression of miR-196a regulates apoptosis, invasion and proliferation in pancreatic cancer cells through ING5.^[54]^ This shows that miRNA research in the direction of pancreatic cancer is gradually becoming mature, with diagnostic and therapeutic research value, which can provide valuable reference for the study of acute pancreatitis.

The third category is the research related to microRNAs that can be used as diagnostic and therapeutic markers for acute pancreatitis. It has been shown that microRNA-146a and microRNA-146b have potential as markers for the management and prognosis of the course of acute pancreatitis;^[55]^ Some scholars performed serum microRNA expression analysis in AP patients and found that miR-92b, miR-10a and miR-7 expression in AP could be used for early diagnosis of AP, and miR-551b-5p could be used to predict the severity of AP;^[56]^ David Goodwin evaluates miR-216a and miR-217 as possible biomarkers for AP injury in rats and mice,^[57]^ selective miRNA expression was found to be a relatively sensitive serum biomarker of pancreatic injury; In addition, miRNAs play a key role in the regulation of gene expression,^[58,59]^ thus, in vitro and in vivo manipulation of miRNA function is a potential therapeutic for modulating disease pathogenesis and significantly facilitates the development of new drugs.

The fourth category is related to the study of non-coding RNA expression in acute pancreatitis. Wang B. et al conducted a comprehensive analysis of the networks of differentially expressed lncRNA, circRNA, and mRNA as well as their competing endogenous RNA (ceRNA) in mice with severe acute pancreatitis,^[60]^ it was discovered that in the ceRNA network, differentially expressed lncRNAs and circRNAs are primarily involved in the apoptotic pathway and calcium signaling pathway; consequently, it is believed that lncRNAs and circRNAs play an important role in the pathogenesis of SAP, which can be used as a starting point to further explore the pathogenesis of SAP and find new targets for SAP; Yansong Xu et al studied the differential expression of miRNAs in severe acute pancreatitis and discovered that complement component 3 is one of the miRNA target genes and associated with SAP, which can be used as an early biomarker of SAP to study the potential pathogenic mechanism of SAP and as a therapeutic target to reduce the incidence or progression of SAP;^[61]^ Caiyun Wen et al analyzed the miRNA expression profile of pancreatitis and screened 40 and 13 differentially expressed miRNAs for pancreatitis, revealing potential miRNA regulatory mechanisms in pancreatitis and identifying useful biomarkers for the diagnosis of pancreatitis;^[62]^ Shishuai Meng’s screening and validation of differentially expressed extracellular miRNAs in acute pancreatitis identified and confirmed the high expression of miR-24 in peripheral blood during AP, indicating that miR-24 may play a role in intercellular communication that contributes to AP-related damage to distant organs;^[21]^ Yuanxu Qu identifies key miRNAs in exosomes of patients with SAP and finds that miR-603, miR-548ad-5p, miR-122-5p, miR-4477a, miR-192-5p, miR-215-5p, miR-583 are positively associated with SAP, which may offer novel insights into the pathogenesis of SAP and function as biomarkers of SAP.^[63]^ Clearly, the analysis of non-coding RNA expression in acute pancreatitis deserves in-depth study by researchers.

The analysis of the keyword outbreak revealed that pancreatic cancer has received widespread attention, which is seen to have important research value and prospects. At the same time, severe acute pancreatitis in acute pancreatitis has also received much attention. As a serious state of acute pancreatitis, severe acute pancreatitis is characterized by acute onset, rapid progression, critical condition, high mortality and many complications,^[64]^ therefore, there is an immediate need to comprehensively analyze the complex pathogenesis and develop evidence-based treatment technology solutions for the core pathology, and non-coding RNA is one of the breakthrough points. The latest keywords to break out in the last 2 years are injury, pathway, and extracellular vesicles, all closely related to non-coding RNAs. The topic of injury has also been mentioned in studies of acute pancreatitis, and acute pancreatitis-associated pulmonary and intestinal injuries have had a large number of correlative studies in recent years,^[65–67]^ It is well illustrated that acute pancreatitis causes multi-organ functional impairment and interacts with each other. Extracellular vehicles (EVs) are generated by cells carrying proteins and nucleic acids that regulate the host immune response by mediating the transfer of intercellular substances and, therefore, play a significant role in the development and progression of inflammatory diseases,^[68]^ and miRNAs have been extensively examined as a key component of EVs in order to investigate their roles in AP development,^[69]^ in addition EVs have other functions that affect acute pancreatitis.^[70]^ And for the keyword “pathway,” which plays a crucial role in the pathogenesis of acute pancreatitis and could function as a biomarker to aid in the diagnosis and treatment of acute pancreatitis in the future.^[71,72]^ In conclusion, keyword burst analysis can objectively forecast future research hotspots and guide researchers in selecting future research directions.

**Figure 9.**
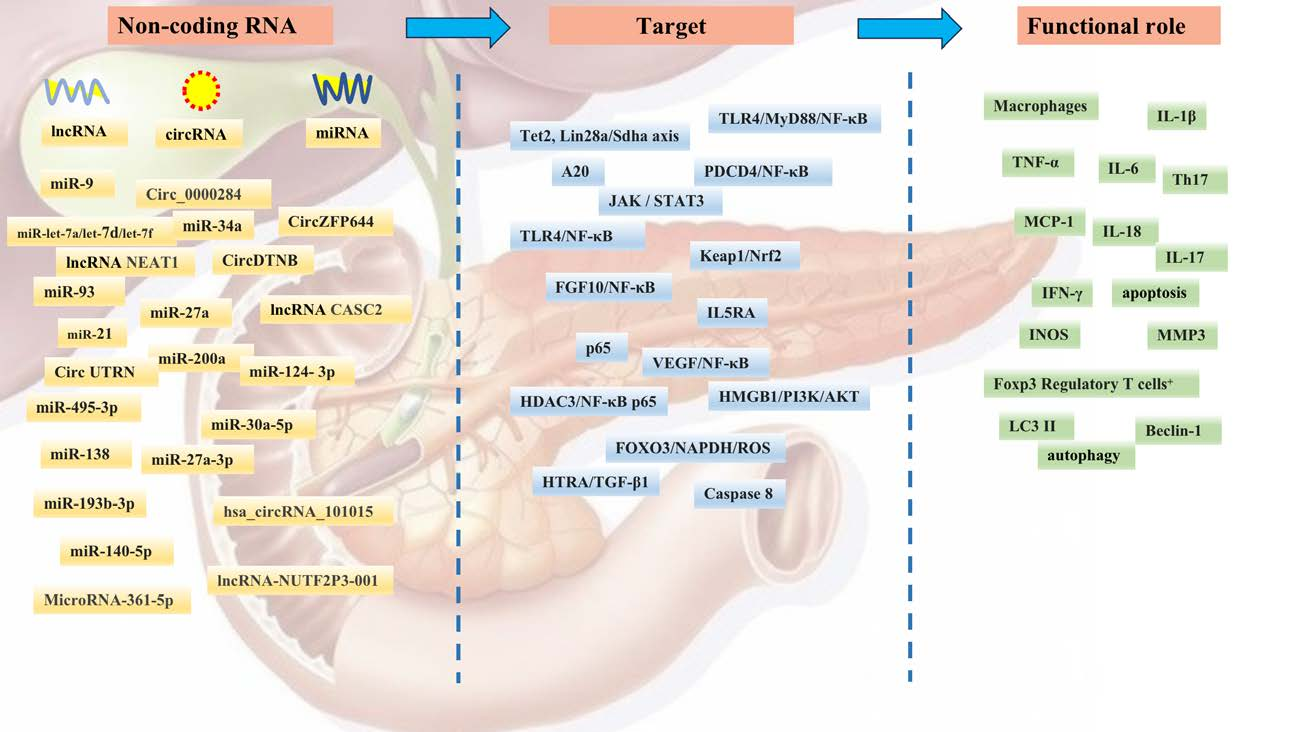
View of the process of non-coding RNA involvement in acute pancreatitis.

In conclusion, non-coding RNAs not only play a role in the occurrence and progression of acute pancreatitis, but they can also be used as diagnostic and therapeutic markers. According to our study, miRNAs were found to be extensively studied, bringing important reference and research value to many researchers, and leading this research field forward, while lncRNAs and circRNAs were relatively less studied and need to be further expanded. Meanwhile, among the 563 literatures in this study, different types of non-coding RNAs were found to be studied by numerous researchers, and we summarized the non-coding RNAs with in-depth studies, as shown in Figure 9, for researchers and experts to discuss and study, hoping to have research value for this research field.

### 4.3. Advantages and limitations

This study is the first to analyze studies in the field of non-coding RNA in acute pancreatitis using a bibliometric system, which can provide a comprehensive guide for scholars who are concerned with this research area. Secondly, in our study, we employed VOSviewer and CiteSpace, which are extensively employed in the field of bibliometrics, and the data involved in the study were all extracted from the WoSCC database, so our data analysis was reliable. Finally, the bibliometric analysis provides a more comprehensive analysis of the hotspots and frontiers of the research field than traditional reviews.

Obviously, this research has some limitations. First, this study relied solely on the WoSCC database and disregarded other databases, which may have led to the omission of pertinent studies. Second, we only examined English-language articles, which may not encompass all studies in this field. Lastly, certain publication types, such as corrections and retractions, may impede data analysis results.

## 5. Conclusion

Non-coding RNAs have important research value and promising applications in acute pancreatitis. The rapid increase in the number of publications in this research area indicates that the study of non-coding RNAs in acute pancreatitis is receiving increasing attention from researchers. Cooperation and communication between nations and institutions must be bolstered to expedite in-depth research in this field. On the one hand, investigating the mechanisms of non-coding RNAs in the development of acute pancreatitis can aid in the analysis of the disease’s specific etiology. On the other hand, non-coding RNAs have significant advantages in the diagnosis and treatment of acute pancreatitis; therefore, the future precision treatment of acute pancreatitis will benefit greatly from the study of non-coding RNA therapeutic strategies. Notably, attention should be paid to the translation of research results, i.e., the clinical application of non-coding RNA in acute pancreatitis, in addition to fundamental research.

## Acknowledgments

We would like to acknowledge everyone who contributed to the study of non-coding RNA in acute pancreatitis.

## Author contributions

**Data curation:** Xiaodong Zhu, Kunrong Liu.

**Formal analysis:** Xiaodong Zhu, Xiping Tang.

**Methodology:** Kunrong Liu, Guozhong Chen.

**Resources:** Guozhong Chen.

**Software:** Xiaodong Zhu.

**Supervision:** Guozhong Chen.

**Writing – original draft:** Xiaodong Zhu.

**Writing – review & editing:** Guozhong Chen.
